# Epidemiology and Mortality of Ovarian Cancer in Taiwan: A Population-Based Study

**DOI:** 10.3390/jcm11195627

**Published:** 2022-09-24

**Authors:** Yun-Hui Teng, Fu-Chao Liu, Shang-Yu Huang, Chang-Fu Kuo, Huang-Ping Yu

**Affiliations:** 1 Department of Anesthesiology, Chang Gung Memorial Hospital, Taoyuan 333, Taiwan; 2 College of Medicine, Chang Gung University, Taoyuan 333, Taiwan; 3 Department of Obstetrics and Gynecology, Chang Gung Memorial Hospital, Taoyuan 333, Taiwan; 4 Division of Rheumatology, Allergy and Immunology, Chang Gung Memorial Hospital, Taoyuan 333, Taiwan; 5 Office for Big Data Research, Chang Gung Memorial Hospital, Taoyuan 333, Taiwan

**Keywords:** ovarian cancer, epidemiology, population-based study, mortality

## Abstract

Ovarian cancer is the second most common cause of death from gynecologic cancer. The aim of this study was to estimate the incidence of ovarian cancer and the trend of mortality in different histological subtypes of ovarian cancer in Taiwan. Patient information regarding ovarian cancer was provided by the Taiwan National Health Insurance database. The histological subtypes of ovarian cancer were retrieved from the Taiwan Cancer Registry database, while the survival rates were extracted from the National Death Registry database. In this population-based cohort study, the annual prevalence, incidence, and overall mortality of ovarian cancer during 2002–2015 were determined. The trend in the incidence and the mortality rate of different histologic subtypes were estimated using joinpoint regression analysis. It was found that age-standardized incidence of ovarian cancer increased from 9.46 in 2002 to 11.92 per 100,000 person-years in 2015, with an average annual percentage change of 2.0 (95% CI = 1.5–2.5). The 1-, 3-, and 5-year mortality rates of overall ovarian cancer declined progressively during the study period, especially the group of Charlson comorbidity index ≤ 1. Ovarian serous carcinoma was the most common histological subtype in Taiwan, comprising 30.9% of ovarian cancer patients in 2002–2015. This study provides valuable information for use in developing healthcare policies for ovarian cancer.

## 1. Introduction

Ovarian cancer is the eighth most frequent cancer diagnosis in the world. Over 314,000 women were diagnosed with ovarian cancer in 2020, of which approximately 207,000 died [1], making it the second most common cause of death from gynecologic cancers.

There are significant geographic differences in ovarian cancer incidence and mortality rates. The regions with a higher reported incidence of ovarian cancer may be associated with a higher prevalence of numerous established risk factors, including nulliparity/low birth rate, menopause hormone therapy use, endometriosis [2], familial genetic predisposition, and lower prevalence of oral contraceptives [3]. Some prior studies have reported on international trends of ovarian cancer incidence, which remain relatively stable. They found significant increases in eastern/southern Europe and Asia (Thailand and Japan) and decreases in northern Europe and North America [2,3]. 

The most common histologic type of ovary cancer is epithelial tumor. Its subtypes include serous, endometrioid, clear cell, mucinous, mixed epithelial, mesenchymal, and other tumors [4,5]. The remaining non-epithelial tumors include sex cord stromal, germ cell, and other tumors [5]. The different subtypes of ovarian cancer are characterized by distinct risk factors, treatment responses, and prognoses. Compared to the global distribution of epithelial subtypes, the distribution of these subtypes does not vary significantly for most countries, with the exception of some countries in Asia [3]. 

Regarding the epidemiology of ovarian cancer in Taiwan, information from global cancer data surveillance and previous studies has not been previously reported. This is the first epidemiological study on ovarian cancer in Taiwan. In this population-based study, we summarize the trends in the incidence of ovarian cancer and provide estimates of ovarian cancer incidence and mortality rate by histologic subtype in Taiwan between 2002 and 2015 by combining information from the National Health Insurance Research Database (NHIRD), Taiwan Cancer Registry Database (TCRD), and National Death Registry (NDR). 

## 2. Materials and Methods

### 2.1. Source Data and Study Population

National Health Insurance (NHI) is a universal health insurance scheme in Taiwan that has provided extensive coverage since 1995, with over 99.6% of citizens being registered beneficiaries. Deidentified computerized data were derived from The Bureau of National Health Insurance to establish the National Health Insurance research database (NHIRD). This NHIRD comprises basic patient information, medical data, dates of admission, operations, and clinical diagnoses. 

This research utilized the NHIRD, Taiwan Cancer Registry Database (TCRD), and National Death Registry (NDR), which are subsets of the NHI database and have been approved by the Department of Statistics, Ministry of Health and Welfare of Taiwan. By using a unique personalized encrypted identifier for each beneficiary, we were able to efficiently cross-link between different subsets of the NHI database. 

This population-based cohort study comprised ethnic Taiwanese patients diagnosed with ovarian cancer (International Classification of Disease for Oncology, Third Edition (ICD-O-3) codes C56, C57.0, C57.1, C57.2, C57.3, and C57.4) identified via the TCRD from 2002 to 2015. Patient information, including age, gender, place of residence, income levels, and occupation, was provided by NHIRD. The histological subtypes of ovarian cancer were retrieved from the TCRD, while the survival rates were extracted from the NDR database.

This epidemiologic study was approved by the Institutional Review Board of Chang Gung Medical Hospital (approval number: 201601233B0D001). The data used in this study were deidentified and encrypted, so the requirement for obtaining patient consent was waived.

### 2.2. Prevalence and Incidence

The crude prevalence rate of ovarian cancer per 100,000 persons was obtained as the number of ovarian cancer patients divided by the total number of women in the specified year. We defined ovarian cancer patients as individuals who were diagnosed with ovarian cancer before 1 July of that calendar year. The total number of women comprised every woman who had registered by 1 July of that calendar year. On the other hand, the crude incidence rate of ovarian cancer per 100,000 person-years refers to the number of new cases of ovarian cancer divided by the total person-years in the at-risk population during a specified year. The new ovarian cancer cases were defined as patients with a record of ovarian cancer in a specified year but without diagnosis of ovarian cancer prior to 1 January of that year. Women with no history of ovarian cancer during the same year were defined as the at-risk population. All eligible subjects were followed up from January 1 of the year in which the earliest diagnosis of ovarian cancer was made until the primary outcome of death or the end of the study period on 31 December 2015.

The geographic variations in the prevalence and incidence of ovarian cancer in 2002 and in 2015 were also compared by dividing Taiwan into 21 administrative districts, including Keelung, Taipei city, New Taipei city, Taoyuan city, Hsinchu, Miaoli, Taichung city, Changhua, Yunlin, Nantou, Chiayi, Tainan city, Kaohsiung city, Pingtung, Yilan, Hualien, Taitung, Lianjiang, and the offshore Penghu islets. The age-standardized prevalence and incidence of ovarian cancer for each district were estimated with reference to the overall number of women in 2015 to diminish the regional effects of diverse age.

### 2.3. Trends of Incidence and Prevalence

The age-standardized prevalence and incidence rate of ovarian cancer in each year from 2002 to 2015 were calculated with reference to the population of women in 2015. In order to determine the trend of incidence, joinpoint regression analysis was used to compare the mean annual percentage change (APC) of the incidence of ovarian cancer during the study period.

### 2.4. Trends of Mortality

Overall mortality of patients with ovarian cancer was extracted from overall mortality rates in the National Death Registry of Taiwan. In the specified calendar year, we calculated 1-, 3-, and 5-year mortality rates for ovarian cancer. Then, our study samples were divided into two groups according to their Charlson comorbidity index (CCI), CCI ≤ 1 or CCI ≥ 2, to compare the trend in mortality rates from 2002 to 2015.

### 2.5. Histological Analysis of Ovarian Cancer Subtypes

Patients with ovarian cancer were analyzed for trends in age, sex, socioeconomic status, incidence, and mortality according to different histological subtypes. The histological subtype of ovarian cancer was categorized according to the classification by World Health Organization (WHO) criteria, with mainly epithelial cancer, germ cell cancer, and other subtypes. Epithelial cancers, which represent the majority of malignant ovarian cancers, were further grouped into the following histological types: serous, mucinous, endometrioid, clear cell, and other [4].

### 2.6. Statistical Analysis

The 95% confidence intervals (95% CIs) for the prevalence, incidence, and mortality rate of ovarian cancer were estimated using Poisson regression. The Joinpoint Regression Program (version 4.0.4; National Cancer Institute, Bethesda, MD, USA) was used to estimate the trends for the prevalence, incidence, and mortality rates of ovarian cancer. The average annual percentage change (APC) was calculated for each linear segment. The Charlson comorbidity index (CCI) score was utilized to evaluate the medical condition and mortality risk of ovarian cancer patients. Statistically, continuous variables of CCI score were compared based on a *t*-test, while categorical variables such as sex and income level were compared through *χ*^2^ analysis. The statistical significance level was set to 0.05. All statistical analyses in this study were calculated using SAS software (version 9.4; SAS Institute, Cary, NC, USA).

## 3. Results

### 3.1. Clinical Characteristics of Patients with Ovarian Cancer

The total eligible population in our study included 23,737,221 beneficiaries enrolled in the National Health Insurance (NHI) in 2015. We identified 15,785 patients with a diagnosis of ovarian cancer between 2002 and 2015. The demographic characteristics of the patients are listed in Table 1. The average age was 52.2 ± 15.3 years. More than half of the patients lived in urban areas (64.6%); 26.3% and 7.6% of the patients lived in suburban and rural areas, respectively. For income levels, the proportion of ovarian cancer was similar in all groups, indicating that there is a weak relationship between the prevalence of ovarian cancer and the level of income in Taiwan. The incident cases of ovarian cancer increased from 816 in 2002 to 1412 in 2015, and the average CCI score at ovarian cancer diagnosis increased from 2.42 ± 3.12 to 4.03 ± 3.45, implying that the relationship between ovarian cancer and other pre-existing medical conditions became stronger.

### 3.2. Prevalence and Incidence of Ovarian Cancer

Table 2 and Table 3 show the prevalence and incidence of ovarian cancer in Taiwan between 2002 and 2015. In 2002, the age-standardized prevalence was 51.7 (95% CI = 50.20–53.32) per 100,000 persons, and then increased yearly to 106.1 (95% CI = 104.27–107.97) per 100,000 persons in 2015. The standardized incidence was 9.46 per 100,000 person-years in 2002, and increased yearly, reaching a peak at 12.46 per 100,000 person-years in 2014. Overall, the age-standardized prevalence of ovarian cancer was 2.05-fold higher in 2015 than in 2002; similarly, the age-standardized incidence of ovarian cancer was 1.26-fold higher in 2015 than in 2002.

**Figure 1:**
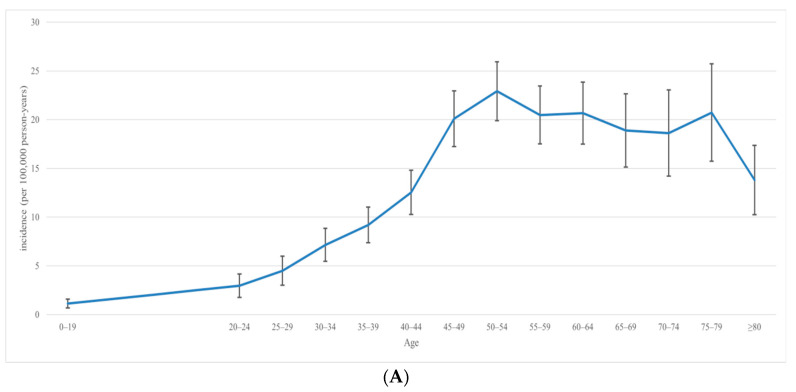

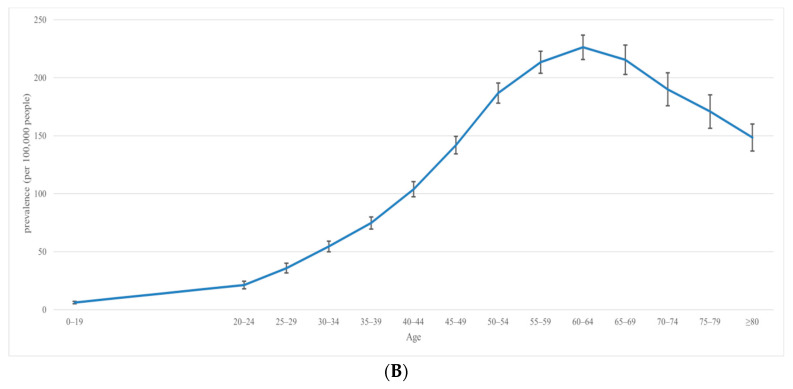
Differences in the trends of (**A**) incidence and (**B**) prevalence of ovarian cancer according to age groups in Taiwan in 2015.

The prevalence and incidence of ovarian cancer in 2015 are shown according to age groups in Figure 1. The age-specific prevalence of ovarian cancer is seen to increase slowly with age, peaking at age 60 and then decreasing, whereas the incidence of ovarian cancer increases slowly with age from a young age, and plateaus after age 45. 

**Figure 2:**
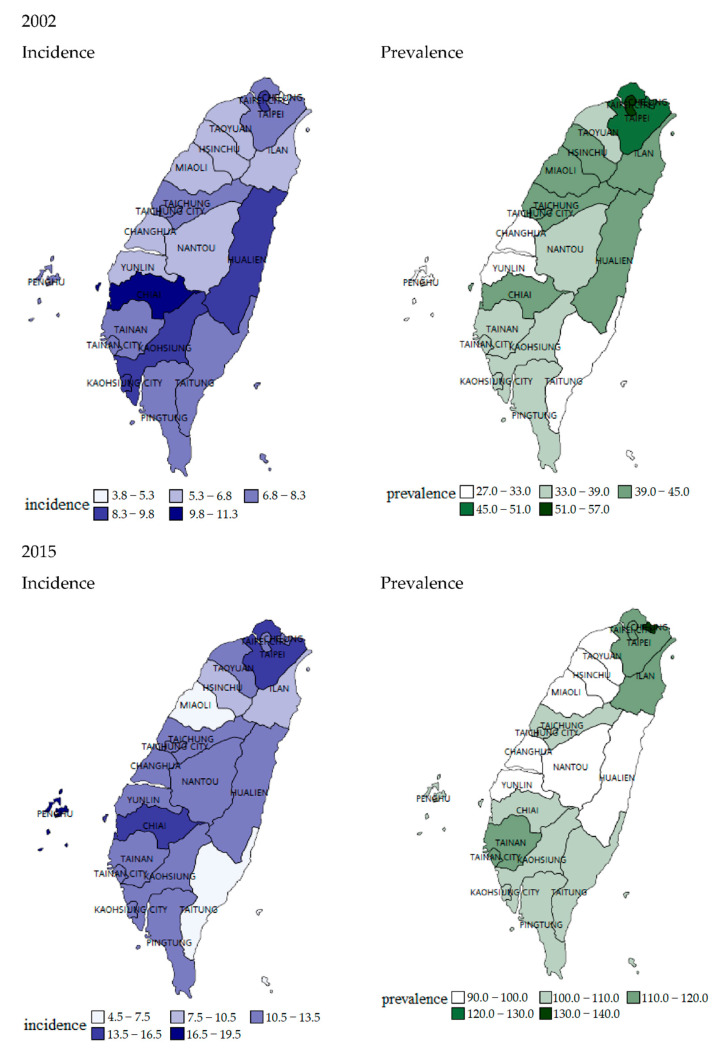
Geographic variation in the prevalence and incidence of ovarian cancer in Taiwan (2002/2015).

Figure 2 shows the geographic distribution of the prevalence and incidence of ovarian cancer in 2002 and 2015. Taipei City, New Taipei city (urban areas), and Chiai (suburban area) had a relatively higher prevalence and incidence than other areas in Taiwan.

### 3.3. Regression Analysis of Prevalence and Incidence of Ovarian Cancer

Table 4 shows the results of joinpoint analysis of the prevalence and incidence of ovarian cancer. The average annual percentage change (APC) in incidence of ovarian cancer was 2.0 per person per year (95% confidence interval, CI: 1.9 to 2.1, *p* < 0.05), which indicates a linear and steady increase in ovarian cancer incidence over the study period.

### 3.4. Ovarian Cancer Mortality Rates

**Figure 3:**
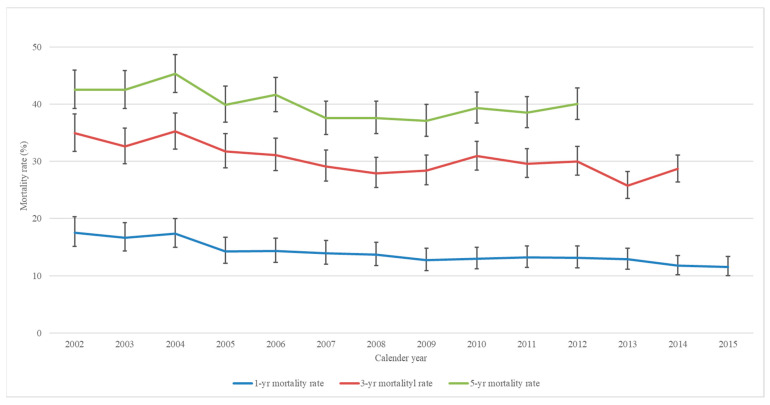
Trends of the one-, two-, and five-year mortality rate of ovarian cancer in Taiwan from 2002 to 2015 (blue: 1-year mortality rate; red: 3-year mortality rate; green: 5-year mortality rate).

Joinpoint analysis of ovarian cancer mortality trends in Taiwan is shown in Table 5. The 1-, 3-, and 5-year mortality rates for ovarian cancer in Taiwan declined progressively during the study period, especially the 1-year mortality rate (Figure 3). Despite these improvements, the 5-year mortality rate in 2012 was still as high as 40.0%. To evaluate the influence of comorbidities on the overall mortality rates, we allocated our study samples into two groups according to their CCI score at ovarian cancer diagnosis. There was a statistically significant difference between the CCI ≤ 1 group and the CCI ≥ 2 groups. In the CCI ≤ 1 group (48.7% of total ovarian cancer patients), the 1-, 3-, and 5-year mortality rates declined significantly year by year. However, in the CCI ≥ 2 group (51.2% of total ovarian cancer patients), only the 1-year mortality rate decreased progressively, whereas the 3- and 5-year mortality rates increased slightly.

### 3.5. Analysis Based on the Histological Subtypes of Ovarian Cancer

Table 6 shows the demographic characteristics of patients with different histological subtypes of ovarian cancer. Of all patients with different histological subtypes, more than 60% lived in urban areas. Patients with ovarian serous carcinoma comprised 30.9% of total ovarian cancer patients during 2002–2015, while germ cell tumor patients accounted for 5.4% of all ovarian cancer patients, which was the lowest percentage observed for histological subtypes. Moreover, patients with serous carcinoma presented at an older age (56.21 ± 12.44 years) and had more comorbidities at diagnosis (CCI: 4.36 ± 3.80) compared to patients with other subtypes of ovarian cancer. On the other hand, patients with germ cell tumors were a younger age at presentation and had fewer comorbidities at diagnosis.

**Table 5:** Joinpoint analysis of ovarian cancer overall mortality ^a^ by Charlson comorbidity index (CCI) in Taiwan from 2002 to 2015.

	Mortality Rate				Average APC	Trend 1		Trend 2		Trend 3	
	2002		End ^b^			Years	APC (95%CI)	Years	APC (95%CI)	Years	APC (95%CI)
Total											
1-year mortality rate	17.52	(15.08–20.31)	11.54	(9.99–13.33)	−2.9 (−3.6 to −2.1) *	2002 to 2015	−2.9 (−3.6 to −2.1) *				
3-year mortality rate	34.93	(31.77–38.31)	28.68	(26.42–31.09)	−1.7 (−2.6 to −0.8) *	2002 to 2014	−1.7 (−2.6 to −0.8) *				
5-year mortality rate	42.52	(39.22–45.99)	40.04	(37.37–42.83)	−1.2 (−2.2 to −0.1) *	2002 to 2012	−1.2 (−2.2 to −0.1) *				
CCI											
CCI ≤ 1											
1-year mortality rate	18.59	(15.10–22.78)	9.13	(6.21–13.31)	−4.8 (−6.5 to −2.9) *	2002 to 2015	−4.8 (−6.5 to −2.9) *				
3-year mortality rate	41.21	(36.55–46.21)	24.50	(19.62–30.33)	−4.6 (−6.2 to −3.0) *	2002 to 2014	−4.6 (−6.2 to −3.0) *				
5-year mortality rate	50.25	(45.45–55.26)	38.17	(32.59–44.34)	−2.7 (−6.4 to 1.2) *	2002 to 2004	5.7 (−11.6 to 26.4)	2004 to 2008	−8.5 (−17.0 to 0.9)	2008 to 2012	−0.7 (−8.9 to 8.3)
CCI ≥ 2											
1-year mortality rate	16.51	(13.28–20.43)	12.10	(10.34–14.13)	−1.8 (−2.5 to −1.1) *	2002 to 2015	−1.8 (−2.5 to −1.1) *				
3-year mortality rate	28.95	(24.85–33.55)	29.55	(27.05–32.22)	0.6 (−0.4 to 1.6)	2002 to 2014	0.6 (−0.4 to 1.6)				
5-year mortality rate	35.17	(30.80–39.95)	40.55	(37.54–43.70)	1.8 (0.9 to 2.7) *	2002 to 2012	1.8 (0.9 to 2.7) *				

APC, annual percent change; CI, confidence interval; CCI, Charlson comorbidity index; * *p* < 0.05. ^a^ Overall mortality of patients with ovarian cancer was extracted from overall mortality rates in the National Death Registry of Taiwan and corresponds to relative mortality rates estimated using the life table method. ^b^ The end year in one-year survival was 2015, the end year in three-year survival was 2014, and the end year in five-year survival was 2012.

**Figure 4:**
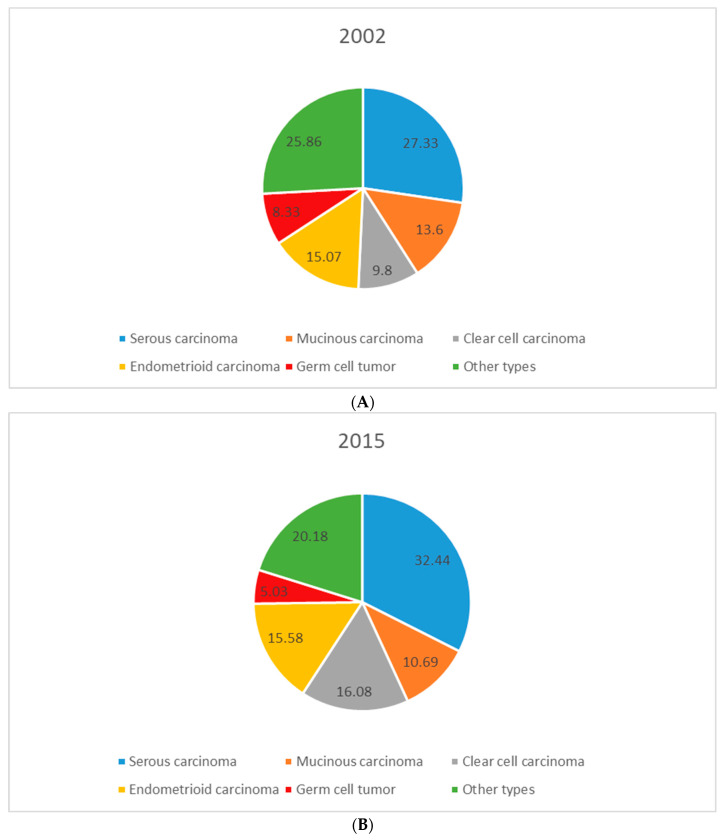
Percentage of different subtypes of ovarian cancer in (**A**) 2002 and (**B**) 2015.

Figure 4 illustrates the incident percentage of different subtypes of ovarian cancer in Taiwan in 2002 and 2015. Serous carcinoma accounted for a slightly higher proportion than other subtypes of ovarian cancer in both 2002 and 2015. The percentage of serous carcinoma and clear cell carcinoma increased substantially during 2002–2015 (serous carcinoma, 27.33% in 2002 to 32.44% in 2015; clear cell carcinoma, 9.8% in 2002 to 16.08% in 2015). Meanwhile, the percentage of mucinous carcinoma decreased slightly during 2002–2015 (13.60% in 2002 to 10.69% in 2015).

Table 7 shows the variation in overall mortality rates for different subtypes of ovarian cancer. In a joinpoint analysis, the 5-year mortality rate in 2012 for serous carcinoma, mucinous carcinoma, clear cell carcinoma, endometrioid carcinoma, germ cell tumor, and other subtypes was 50.42%, 25.95%, 35.71%, 25.02%, 6.06%, and 54.00%, respectively. 

**Figure 5:**
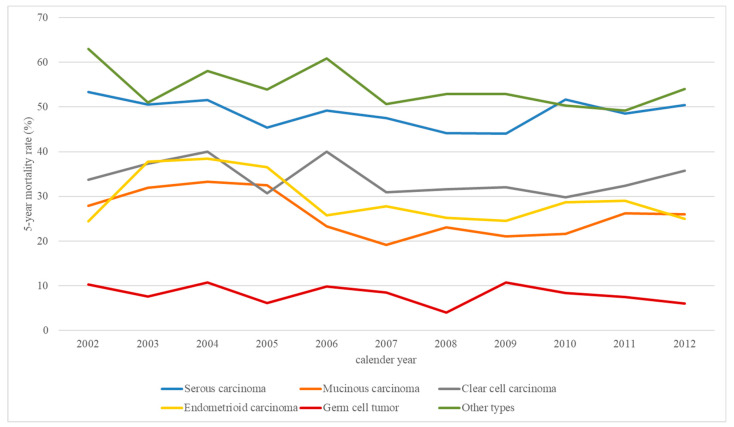
Trends of the 5-year mortality rate of patients with different types of ovarian cancer.

The secular trends of 5-year overall mortality for patients with different subtypes (Figure 5) revealed that serous carcinoma had relatively poor prognosis, and germ cell tumor had a relatively lower mortality rate. 

## 4. Discussion

In this population-based cohort study, the temporal change in the incidence of ovarian cancer and the mortality rate by histologic subtype in Taiwan between 2002 and 2015 were evaluated. Ovarian cancer incidence rates vary depending on geographical region. International ovarian incidence and temporal change from the 1970s to 2000s have been reported in a previous study [3]. During the 5-year period of the study (2003–2007), the incidence of ovarian cancer was highest in Europe, especially in eastern and southern Europe. Intermediate rates of incidence were found in North and South America, while the lowest incidence was seen in Oceania and Asia, including Singapore, Thailand, Japan, South Korea, and Hong Kong [3,6,7]. 

In our study, the age-standardized incidence rate of ovarian cancer in Taiwan was 9.46 per 100,000 person-years in 2002, which is lower than the reported incidence rate in Europe but still higher than North/South America and the Asia regions mentioned above. There was some variation in the incidence over time within specific regions. From the 1970s to the 2000s, the incidence of ovarian cancer decreased in North America and Northern Europe, while it increased gradually in Eastern/Southern Europe and Asia, such as in Japan, South Korea, and Hong Kong. In Taiwan, the trend of incidence was stable, rising with an average APC of 2.0 (95% CI 1.9–2.1) during 2002–2015. A similar rising trend was observed for other countries in Asia.

Many studies have concluded that oral contraceptive (OC) use is inversely associated with ovarian cancer risk [8]; it is reduced by nearly 30% among regular oral contraceptive users compared with those who had never used oral contraceptives [9]. According to the survey conducted by the government in Taiwan, from 2004 to 2016, the percentage of reproductive aged women who had ever used oral contraceptives was only 5.41% to 7.94% [10]. In European and North American countries, the percentage of those that had ever used oral contraceptives is 31 to 37% [9], which is much higher than in Taiwan. 

Nulliparity and low parity are associated with increased risk of ovarian cancer [11]. Women who have ever given birth have a reduced risk of ovarian cancer, and for each additional birth the risk is further reduced by 10–20% [8,11]. This effect appears in all major histotypes of epithelial ovarian cancer but is probably strongest for clear cell and endometrioid carcinomas [12]. Taiwan is one of the regions with the low birth rates in the world, and from 2002 to 2015, the crude birth rate dropped from 11.02 to 9.10‰ [13]. This may be one of the possible reasons for the increasing incidence of ovarian cancer in Taiwan.

In Europe and North America, the proportion of ovarian cancers corresponding to serous carcinoma is usually over 40%, and the proportion corresponding to clear cell carcinoma is normally less than 10% [3]. However, in our study, patients with ovarian serous carcinoma comprised 30.9% of total ovarian cancer patients during 2002–2015, and clear cell carcinoma patients accounted for 13.6% of all ovarian cancer patients, which is similar to representations reported for other Asian countries such as Japan, Singapore, and Thailand [2,3]. The variation in the distribution of epithelial histologies may be explained by population differences in genetic and environmental risk factors, which have different effects on each histologic subtype [3]. In terms of genetic factors, the role of BRCA1 and BRCA2 mutations as risk factors for serous carcinomas has been established [14,15]. BRCA1/2 mutations are common in Taiwanese patients with serous ovarian carcinoma, and similar mutation rates are observed in other ethnic groups [16]. However, differences in BRCA1 or BRCA2 mutation rates are unlikely to explain the low incidence of serous tumors in Asian countries [2]. 

Other risk factors, such as endometriosis, may also affect the distribution of certain histological subtypes. Recent molecular and pathological studies suggest that endometriosis may be a precursor to ovarian cancer, particularly endometrioid and clear cell ovarian cancer [17]. The proportion of clear cell carcinoma and endometrioid carcinoma has been increasing in Japan and Thailand relative to the international distribution of these subtypes [3]. Part of the reason for this may be that the incidence of endometriosis is slightly higher among women of Asian ethnicity [18,19]. Moreover, in our study, the percentage of clear cell carcinoma increased substantially from 9.8% to 16.08% during 2002–2015. However, no trend of endometriosis incidence has been reported in the last few decades in Taiwan, so it is not possible to determine whether it is associated with the increasing proportion of clear cell carcinoma. Therefore, more attention and further research should be given to the etiological factors of histology-specific ovarian cancer.

Several population-based studies have reported that the survival trend of ovarian cancer patients has gradually improved over time [7,20,21,22], although there has been some variation regarding the improvement of survival rates across areas. Our study showed a trend toward improved survival for ovarian cancer in Taiwan between 2002 and 2015. The mortality rates of ovarian cancer in Taiwan progressively declined during the study period, especially for 1-year mortality. In addition to increased medical accessibility, improvements in general healthcare, more aggressive surgical treatment, and advances in chemotherapy have contributed to an improved survival trend during this period. However, in our study, the 1-year mortality rate showed greater improvement than the 5-year mortality rate. According to the American Cancer Society (2019), early detection of ovarian cancer at a localized stage (Stages 1A and 1B) results in far better disease prognosis [23]. Therefore, our results show that, from 2002 to 2015, progress in early diagnosis was limited. After further analysis, the mortality rate of the CCI ≤ 1 group was significantly less than that of CCI ≥ 2 group. CCI was found to be a very important prognostic factor in the 1-, 3-, and 5-year mortality rate. 

Compared with other reported population-based series after 2002, the 5-year relative survival rates of ovarian cancer patients in our study were comparable to those reported in other developed urban areas, such as Hong Kong [7]. According to previous reports, the 5-year relative survival rate of ovarian cancer patients in urban areas of developed Asian populations such as Singapore [24], South Korea, and Turkey [25] was higher than in less developed countries such as Thailand and India [25]. As in other developed regions in Asia, high accessibility to medical services and availability of diagnostic facilities in Taiwan could partially explain the survival rates being higher than in less-developed regions in Asia. In addition to the impact of medical accessibility, the prognostic factors of ovarian cancer are also related to subtype and stage at the time of diagnosis. Serous carcinoma is the subtype of ovarian cancer with the lowest five-year survival rate and for which the least improvement in survival from 2002 to 2015 was observed. Delayed diagnosis is one of the major reasons for the poor long-term survival rate of ovarian cancer patients, as most patients are at an advanced stage at the time of first diagnosis [23]. Therefore, early diagnosis of serous carcinoma is the main goal of future efforts.

There were several limitations of our research. First, clinical information on stages and treatment of ovarian cancer was not presented in this study due to limitations of the available databases with respect to this information. Therefore, we were unable to assess the possible impact of early surveillance, new therapies, and other prognostic factors on the overall mortality of ovarian cancer. Nevertheless, we managed to incorporate the latest evidence and conduct a comprehensive epidemiological investigation of ovarian cancer in Taiwan. Second, the relationship between known risk factors and the incidence or mortality rate of ovarian cancer is difficult to assess based on the available information. For example, evidence from a previous analysis has indicated that current use of menopausal hormone therapy (MHT) increases the risk of ovarian cancer, and that even after stopping, the risk remains elevated in women who used MHT for five or more years [26]. From the database, only the percentage of menopausal hormone therapy in a given year and the trend of change over a period of time could be extracted and not the detailed dose or exact duration of use. Third, we could not discern the complete personal habits of each patient from the NHIRD. Thus, lack of information on risk factors such as smoking habits, family history, fat intake, environmental risk factors, and alcohol consumption [8] is also a limitation of this study. However, despite these limitations, the data provided by the National Health Bureau are generally accurate and provide nationwide coverage. Therefore, our study may help researchers to understand the epidemiological status of ovarian cancer in Taiwan and allow comparison of the findings with those based on populations in other parts of the world.

## 5. Conclusions

In summary, the major strength of this study is the large sample size selected from a population-based database rather than a single institution, providing the first report on ovarian cancer epidemiology in Taiwan. Our results found both the prevalence and incidence of ovarian cancer gradually increased in Taiwan from 2002 to 2015. The mortality rate was seen to slightly decline in our sample of ovarian cancer patients during the study period. The epidemiological report presented here provides valuable information for the development of future healthcare policies aimed at reducing the incidence and mortality of ovarian cancer. More resources should be dedicated to improving diagnosis through early surveillance and developing effective treatments for ovarian cancer patients.

## Figures and Tables

**Table 1:** Clinical characteristics of ovarian cancer patients in Taiwan from 2002 to 2015.

	Entire Cohort (*n* = 15,785)		By Calendar Year				
			2002 (*n* = 816)		2015 (*n* = 1412)		*p* Value
Age (years) (mean ± standard deviation)	52.29 ± 15.35		51.72 ± 16.01		53.07 ± 14.95		0.0505
Place of residence, No. (%)							0.0005 *
Urban	10,202	(64.63)	532	(65.20)	926	(65.58)	
Suburban	4157	(26.34)	199	(24.39)	380	(26.91)	
Rural	1200	(7.60)	71	(8.70)	103	(7.29)	
Unknown	226	(1.43)	14	(1.72)	3	(0.21)	
Income levels, No. (%)							<0.0001 *
Quintile 1	3118	(19.75)	142	(17.40)	255	(18.06)	
Quintile 2	2982	(18.89)	393	(48.16)	132	(9.35)	
Quintile 3	3137	(19.87)	20	(2.45)	352	(24.93)	
Quintile 4	3183	(20.16)	120	(14.71)	325	(23.02)	
Quintile 5	3177	(20.13)	132	(16.18)	347	(24.58)	
Unknown	188	(1.19)	9	(1.10)	1	(0.07)	
Occupation, No. (%)							<0.0001 *
Dependents of the insured individuals	4786	(30.32)	268	(32.84)	447	(31.66)	
Civil servants, teachers, military personnel, and veterans	899	(5.70)	39	(4.78)	74	(5.24)	
Nonmanual workers and professionals	3447	(21.84)	144	(17.65)	366	(25.92)	
Manual workers	4861	(30.80)	295	(36.15)	372	(26.35)	
Other	1792	(11.35)	70	(8.58)	153	(10.84)	
Charlson index (mean ± standard deviation)	3.44 ± 3.43		2.42 ± 3.12		4.03 ± 3.45		<0.0001 *

* *p* < 0.05.

**Table 2:** Crude and age-standardized prevalence (per 100,000 people) of ovarian cancer in Taiwan from 2002 to 2015.

Year	Prevalence					
	Number of Cases	Taiwan Female Population	Crude		Standardized	
2002	4664	11,352,804	41.08	(39.90–42.26)	51.76	(50.20–53.32)
2003	5122	11,418,978	44.86	(43.63–46.08)	55.60	(54.01–57.19)
2004	5623	11,475,713	49.00	(47.72–50.28)	59.87	(58.24–61.50)
2005	6140	11,528,971	53.26	(51.93–54.59)	63.87	(62.22–65.53)
2006	6669	11,577,930	57.60	(56.22–58.98)	67.78	(66.10–69.46)
2007	7273	11,625,350	62.56	(61.12–64.00)	72.32	(70.62–74.03)
2008	7895	11,670,723	67.65	(66.16–69.14)	76.62	(74.89–78.35)
2009	8510	11,711,832	72.66	(71.12–74.21)	80.63	(78.88–82.37)
2010	9167	11,746,408	78.04	(76.44–79.64)	85.06	(83.30–86.82)
2011	9871	11,775,009	83.83	(82.18–85.48)	89.55	(87.77–91.33)
2012	10,534	11,821,126	89.11	(87.41–90.81)	93.60	(91.81–95.40)
2013	11,194	11,870,801	94.30	(92.55–96.05)	97.50	(95.69–99.31)
2014	11,882	11,904,725	99.81	(98.01–101.60)	101.47	(99.65–103.30)
2015	12,676	11,945,052	106.12	(104.27–107.97)	106.12	(104.27–107.97)

**Table 3:** Crude and age-standardized incidence (per 100,000 person-years) of ovarian cancer in Taiwan from 2002 to 2015.

	Incidence					
	Number of Cases	Taiwan Female Population	Crude		Standardized	
2002	816	11,233,179	7.26	(6.77–7.76)	9.46	(8.78–10.14)
2003	859	11,305,581	7.60	(7.09–8.11)	9.48	(8.82–10.14)
2004	883	11,370,918	7.77	(7.25–8.28)	9.71	(9.05–10.38)
2005	932	11,426,403	8.16	(7.63–8.68)	9.69	(9.05–10.33)
2006	1027	11,479,273	8.95	(8.40–9.49)	10.48	(9.82–11.14)
2007	1071	11,527,754	9.29	(8.73–9.85)	10.62	(9.97–11.27)
2008	1120	11,573,487	9.68	(9.11–10.24)	10.87	(10.22–11.52)
2009	1141	11,617,937	9.82	(9.25–10.39)	10.74	(10.11–11.37)
2010	1264	11,658,091	10.84	(10.24–11.44)	11.74	(11.09–12.40)
2011	1243	11,688,571	10.63	(10.04–11.23)	11.23	(10.61–11.86)
2012	1239	11,718,184	10.57	(9.98–11.16)	11.04	(10.42–11.66)
2013	1331	11,763,497	11.31	(10.71–11.92)	11.62	(11.00–12.25)
2014	1447	11,808,153	12.25	(11.62–12.89)	12.42	(11.78–13.06)
2015	1412	11,841,957	11.92	(11.30–12.55)	11.92	(11.30–12.55)

**Table 4:** Joinpoint analysis of ovarian cancer prevalence and incidence in Taiwan from 2002 to 2015.

	Ovarian Cancer Prevalence (per 100,000 People)				Average APC	Trend 1		Trend 2		Trend 3	
	2002		2015			Years	APC (95%CI)	Years	APC (95%CI)	Years	APC (95%CI)
Prevalence	51.76	(50.20–53.32)	106.12	(104.27–107.97)	5.6 (5.4–5.8) *	2002–2007	6.9 (6.5–7.2) *	2007–2011	5.4 (4.8–6.0) *	2011–2015	4.3 (4.0–4.6) *
Incidence	9.46	(8.78–10.14)	11.92	(11.30–12.55)	2.0 (1.5–2.5) *	2002–2015	2.0 (1.5–2.5) *				
Prevalence						Incidence					
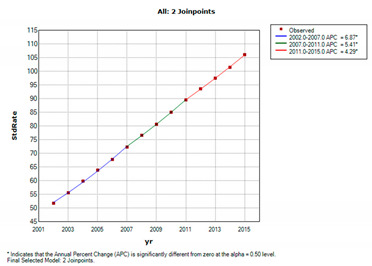						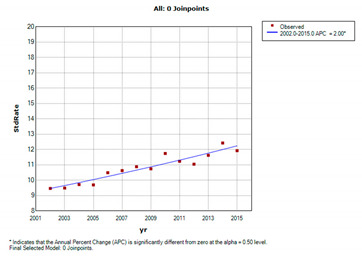					

APC, annual percent change; CI, confidence interval; * *p* < 0.05.

**Table 6:** Clinical characteristics of ovarian cancer patients with different histological subtypes in Taiwan from 2002 to 2015.

	Serous Carcinoma *N* = 4751		Mucinous Carcinoma *N* = 2029		Clear Cell Carcinoma *N* = 2161		Endometrioid Carcinoma *N* = 2211		Germ Cell Tumor *N* = 867		Other Types *N* = 3769	
Age (years) (mean ± standard deviation)	56.21 ± 12.44		47.89 ± 16.09		50.43 ± 9.50		51.00 ± 12.02		27.26 ± 14.33		57.26 ± 16.38	
Place of residence, No. (%)												
Urban	3045	(64.09)	1272	(62.69)	1525	(70.57)	1502	(67.93)	537	(61.94)	2322	(61.61)
Suburban	1269	(26.71)	563	(27.75)	516	(23.88)	555	(25.10)	253	(29.18)	1002	(26.59)
Rural	366	(7.70)	161	(7.93)	98	(4.53)	130	(5.88)	62	(7.15)	383	(10.16)
Unknown	71	(1.49)	33	(1.63)	22	(1.02)	24	(1.09)	15	(1.73)	62	(1.64)
Income levels, No. (%)												
Quintile 1	921	(19.39)	393	(19.37)	426	(19.71)	414	(18.72)	139	(16.03)	825	(21.89)
Quintile 2	855	(18.00)	424	(20.90)	315	(14.58)	407	(18.41)	210	(24.22)	772	(20.48)
Quintile 3	1023	(21.53)	396	(19.52)	355	(16.43)	429	(19.40)	165	(19.03)	770	(20.43)
Quintile 4	947	(19.93)	426	(21.00)	470	(21.75)	465	(21.03)	187	(21.57)	688	(18.25)
Quintile 5	951	(20.02)	360	(17.74)	576	(26.65)	475	(21.48)	154	(17.76)	661	(17.54)
Unknown	54	(1.14)	30	(1.48)	19	(0.88)	21	(0.95)	12	(1.38)	53	(1.41)
Occupation, No. (%)												
Dependents of the insured individuals	1537	(32.35)	582	(28.68)	486	(22.49)	504	(22.80)	431	(49.71)	1248	(33.11)
Civil servants, teachers, military personnel, and veterans	251	(5.28)	101	(4.98)	183	(8.47)	147	(6.65)	19	(2.19)	198	(5.25)
Nonmanual workers and professionals	890	(18.73)	550	(27.11)	610	(28.23)	565	(25.55)	198	(22.84)	634	(16.82)
Manual workers	1591	(33.49)	533	(26.27)	611	(28.27)	740	(33.47)	133	(15.34)	1253	(33.24)
Other	482	(10.15)	263	(12.96)	271	(12.54)	255	(11.53)	86	(9.92)	436	(11.57)
Charlson index (mean ± standard deviation)	4.36 ± 3.80		2.54 ± 2.72		2.73 ± 2.67		3.24 ± 3.12		2.13 ± 2.37		3.60 ± 3.70	

**Table 7:** Joinpoint analysis of overall mortality ^a^ for ovarian cancer patients with different histological subtypes in Taiwan during 2002–2015.

	Ovarian Cancer Mortality Rate				Average APC	Trend 1		Trend 2		Trend 3	
	2002		End ^b^			Years	APC (95%CI)	Years	APC (95%CI)	Years	APC (95%CI)
Serous carcinoma											
1-year mortality rate	13.45	(9.61–18.67)	9.39	(7.05–12.45)	−2.1 (−5.1 to 1.0)	2002 to 2015	−2.1 (−5.1 to 1.0)				
3-year mortality rate	40.36	(34.26–47.11)	30.08	(26.20–34.39)	−1.2 (−3.2 to 1.0)	2002 to 2014	−1.2 (−3.2 to 1.0)				
5-year mortality rate	53.36	(46.98–60.03)	50.42	(45.50–55.55)	−0.4 (−1.7 to 1.0)	2002 to 2012	−0.4 (−1.7 to 1.0)				
Mucinous carcinoma											
1-year mortality rate	14.41	(9.09–22.45)	8.61	(5.09–14.36)	−3.8 (−8.4 to 0.9)	2002 to 2015	−3.8 (−8.4 to 0.9)				
3-year mortality rate	26.13	(18.95–35.36)	18.75	(13.51–25.71)	−3.4 (−6.5 to −0.1) *	2002 to 2014	−3.4 (−6.5 to −0.1) *				
5-year mortality rate	27.93	(20.54–37.28)	25.95	(19.82–33.53)	−0.7 (−8.6 to 7.8)	2002 to 2004	12.5 (−21.8 to 61.8)	2004 to 2007	−17.3 (−41.9 to 17.8)	2007 to 2012	5.3 (−2.7to 14.1)
Clear cell carcinoma											
1-year mortality rate	11.25	(6.02–20.50)	7.93	(5.07–12.29)	−4.2 (−6.5 to −1.8) *	2002 to 2015	−4.2 (−6.5 to −1.8) *				
3-year mortality rate	23.75	(15.86–34.67)	28.99	(23.67–35.21)	−1.1 (−3.5 to 1.4)	2002 to 2014	−1.1 (−3.5 to 1.4)				
5-year mortality rate	33.75	(24.54–45.23)	35.71	(29.45–42.85)	−1.2 (−3.4 to 1.2)	2002 to 2012	−1.2 (−3.4 to 1.2)				
Endometrioid carcinoma											
1-year mortality rate	6.50	(3.31–12.58)	5.45	(3.13–9.41)	−2.2 (−6.3 to 2.1)	2002 to 2015	−2.2 (−6.3 to 2.1)				
3-year mortality rate	17.89	(12.16–25.88)	21.30	(16.41–27.38)	−2.1 (−4.6 to 0.3)	2002 to 2014	−2.1 (−4.6 to 0.3)				
5-year mortality rate	24.39	(17.74–32.99)	25.02	(19.09–32.39)	−2.9 (−6.2 to 0.5)	2002 to 2012	−2.9 (−6.2 to 0.5)				
Germ cell tumor											
1-year mortality rate	4.41	(1.44–13.06)	2.82	(0.71–10.80)	NA	2002 to 2015	NA				
3-year mortality rate	10.29	(5.05–20.38)	11.29	(5.55–22.23)	−1.2 (−5.4 to 3.3)	2002 to 2014	−1.2 (−5.4 to 3.3)				
5-year mortality rate	10.29	(5.05–20.38)	6.06	(2.32–15.35)	−2.8 (−7.7 to 2.5)	2002 to 2012	−2.8 (−7.7 to 2.5)				
Other subtypes											
1-year mortality rate	36.49	(30.40–43.38)	26.32	(21.60–31.84)	−1.8 (−2.9 to −0.6) *	2002 to 2015	−1.8 (−2.9 to −0.6) *				
3-year mortality rate	55.92	(49.37–62.70)	40.83	(35.42–46.73)	−1.8 (−2.8 to −0.8) *	2002 to 2014	−1.8 (−2.8 to −0.8) *				
5-year mortality rate	63.03	(56.55–69.51)	54.00	(48.24–59.97)	−1.5 (−3.0 to−0.0) *	2002 to 2012	−1.5 (−3.0 to 0.0) *				

APC, annual percent change; CI, confidence interval; NA, not available; * *p* < 0.05. ^a^ Overall mortality of patients with ovarian cancer was extracted from overall mortality rates in the National Death Registry of Taiwan and corresponds to relative mortality rates estimated using the life table method. ^b^ The end year in one-year survival was 2015, the end year in three-year survival was 2014, and the end year in five-year survival was 2012.

## Data Availability

The data used to support the findings of this study are available from the corresponding author upon request.
